# Evaluation of an artificial intelligence coronary artery calcium scoring model from computed tomography

**DOI:** 10.1007/s00330-022-09028-3

**Published:** 2022-08-20

**Authors:** Abdul Rahman Ihdayhid, Nick S. R. Lan, Michelle Williams, David Newby, Julien Flack, Simon Kwok, Jack Joyner, Sahil Gera, Lawrence Dembo, Brendan Adler, Brian Ko, Benjamin J. W. Chow, Girish Dwivedi

**Affiliations:** 1 Department of Cardiology, Fiona Stanley Hospital, Perth, Australia; 2 Harry Perkins Institute of Medical Research, Curtin University, Perth, Australia; 3 Harry Perkins Institute of Medical Research, University of Western Australia, Perth, Australia; 4 Centre for Cardiovascular Science, University of Edinburgh, Edinburgh, Scotland UK; 5 Artrya, Perth, Australia; 6 Envision Medical Imaging, Perth, Australia; 7 Monash Cardiovascular Research Centre, Monash University and MonashHeart, Monash Health, Melbourne, Australia; 8 University of Ottawa Heart Institute, Ottawa, ON Canada

**Keywords:** Coronary artery disease, Tomography, X-ray computed, Artificial intelligence, Cardiovascular diseases, Neural networks

## Abstract

**Objectives:**

Coronary artery calcium (CAC) scores derived from computed tomography (CT) scans are used for cardiovascular risk stratification. Artificial intelligence (AI) can assist in CAC quantification and potentially reduce the time required for human analysis. This study aimed to develop and evaluate a fully automated model that identifies and quantifies CAC.

**Methods:**

Fully convolutional neural networks for automated CAC scoring were developed and trained on 2439 cardiac CT scans and validated using 771 scans. The model was tested on an independent set of 1849 cardiac CT scans. Agatston CAC scores were further categorised into five risk categories (0, 1–10, 11–100, 101–400, and > 400). Automated scores were compared to the manual reference standard (level 3 expert readers).

**Results:**

Of 1849 scans used for model testing (mean age 55.7 ± 10.5 years, 49% males), the automated model detected the presence of CAC in 867 (47%) scans compared with 815 (44%) by human readers (*p* = 0.09). CAC scores from the model correlated very strongly with the manual score (Spearman’s *r* = 0.90, 95% confidence interval [CI] 0.89–0.91, *p* < 0.001 and intraclass correlation coefficient = 0.98, 95% CI 0.98–0.99, *p* < 0.001). The model classified 1646 (89%) into the same risk category as human observers. The Bland–Altman analysis demonstrated little difference (1.69, 95% limits of agreement: −41.22, 44.60) and there was almost excellent agreement (Cohen’s *κ* = 0.90, 95% CI 0.88–0.91, *p* < 0.001). Model analysis time was 13.1 ± 3.2 s/scan.

**Conclusions:**

This artificial intelligence–based fully automated CAC scoring model shows high accuracy and low analysis times. Its potential to optimise clinical workflow efficiency and patient outcomes requires evaluation.

**Key Points:**

*• Coronary artery calcium (CAC) scores are traditionally assessed using cardiac computed tomography and require manual input by human operators to identify calcified lesions.*

*• A novel artificial intelligence (AI)–based model for fully automated CAC scoring was developed and tested on an independent dataset of computed tomography scans, showing very high levels of correlation and agreement with manual measurements as a reference standard.*

*• AI has the potential to assist in the identification and quantification of CAC, thereby reducing the time required for human analysis.*

**Supplementary Information:**

The online version contains supplementary material available at 10.1007/s00330-022-09028-3.

## Introduction

Coronary artery disease (CAD) is a leading cause of death and disability worldwide, which imposes a substantial burden on healthcare expenditure [1]. Early identification of asymptomatic individuals at high cardiovascular risk is important for optimising the use of preventive pharmacotherapies, such as statins. The coronary artery calcium (CAC) score is a surrogate measure of atherosclerotic plaque burden within the coronary arteries that has been shown to predict CAD events [2, 3]. CAC is traditionally assessed non-invasively using electrocardiogram-gated computed tomography (CT) imaging of the heart, without the need for intravenous contrast [3]. Uptake in clinical practice is likely to increase, as guidelines now recommend CAC scoring for reclassifying cardiovascular risk in intermediate or borderline risk individuals, which thereby assists in decision-making around preventive therapies [4–6].

CAC is currently assessed manually by radiographers using CT image axial slices and then quantified using commercially available semi-automatic software. The radiographer must select high-density voxels, defined as > 130 Hounsfield units (HU), which are identified semi-automatically and then segmented according to their location in the coronary arteries.[3] Importantly, the radiographer must inspect the location of high-density voxels to exclude non-coronary calcification. CAC is usually quantified using the Agatston method, which considers calcified plaque area and maximal density of individual calcified lesions, but not location and distributional pattern [7, 8]. The Agatston score can be categorised into cardiovascular risk categories based on the score: 0 (no CAC), 1–10 (minimal CAC), 11–100 (mild CAC), 101–400 (moderate CAC), and > 400 (severe CAC) [3, 9]. CAC scoring, albeit straightforward, adds to costs and can be time-consuming and impractical for large-scale studies.

Automated models for CAC assessment using cardiac CT have been reported, including rule-based, machine learning, and deep learning approaches [10–20]. A challenge for fully automated scoring methods is the ability to discriminate between true CAC and calcification in surrounding structures, such as that of the mitral annulus, heart valves, and the aorta. Despite promising results, these automated methods have not yet been adopted into routine clinical practice as more evidence is necessary and generalisability needs to be demonstrated. Fully automated identification and quantification of CAC that requires very little or no human analysis can potentially decrease the workload of human operators, be more time-efficient, reduce costs, and play a role in large-scale screening strategies or epidemiological studies [21–23]. This study aims to develop and evaluate a novel artificial intelligence (AI)–based fully automated model that identifies and quantifies CAC using cardiac CT.

## Material and methods

All CT scans used for training, validation, and testing of the automated CAC scoring model (DeepC Architecture, Salix, Artrya Ltd.) were retrospectively obtained. This study was approved by the local research ethics committee (Bellberry Human Research Ethics Committee: 2020-06-533) and was conducted in accordance with the Declaration of Helsinki.

### Model development

**Fig. 1:**
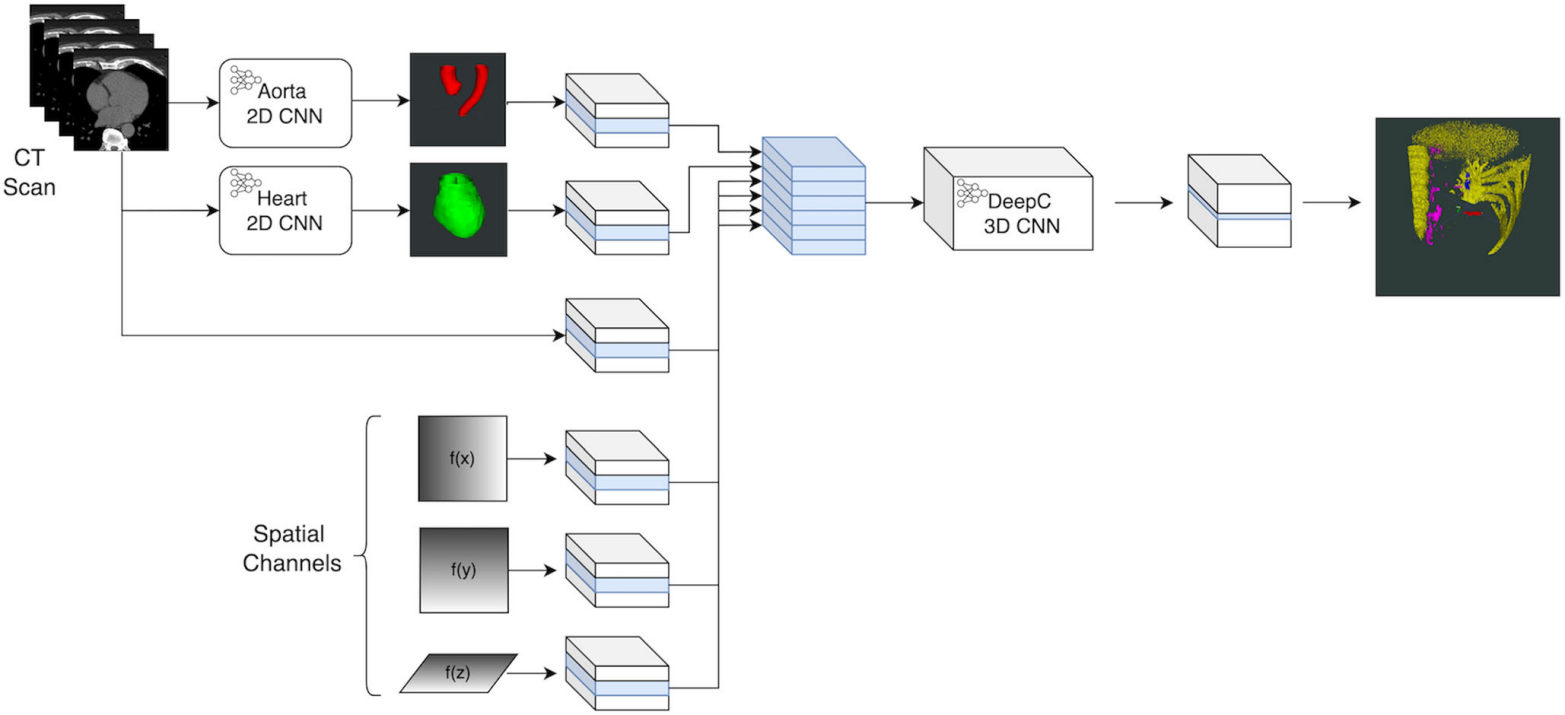
Architecture diagram for the automated CAC scoring algorithm. Abbreviations: CAC coronary artery calcium, CNN convolutional neural networks, CT computed tomography. Figure legend: As a pre-processing step, two-dimensional (2D) convolutional neural networks (CNN) are used to segment the ascending and descending aorta, and heart area. The 2D CNNs process the computed tomography (CT) scan slice by slice in the axial direction to generate a three-dimensional (3D) segmentation. The DeepC 3D CNN combines six input channels, each representing a 3D sub-volume. Of these six input channels, two are used for the aorta and heart segmentation, and a further three to provide spatial context relative to the centre of the scan in the x, y, and z directions. The final channel represents the Hounsfield unit (HU) subvolume. The extended input channels enable the CNN to classify specific HU patterns based on where they occur spatially in the scan. The 3D CNN then labels each coronary artery and an Agatston CAC score is calculated

A three-dimensional (3D) fully convolutional neural network (CNN) was developed to classify high-density voxels (defined as >130 HU) in the non-contrast CT volume as shown in Fig. 1. This custom CNN is a 13-layer model consisting of 3D convolution layers. The CNN field of view comprises an area of 33 mm in the z direction and 60.5 mm in the axial plane, which provides the spatial context the CNN uses to predict the classification of lesions identified. A separate U-NET-based CNN was developed to identify the ascending and descending aorta from the CT axial slices. The output of the aortic segmentation was used as an additional input channel to the CAC scoring CNN. A second two-dimensional CNN, with a similar design to the aortic segmentation model, was used to segment the cardiac area of interest from the scan. The use of these segmentation models was incorporated to reduce false positives by eliminating areas of the scan with non-coronary calcifications.

A unique aspect of the model is the use of additional input channels to the CNN to provide spatial location information relative to a spatial reference point in the scan. As the CNN only has access to a subset of the CT volume when inferencing (i.e., the field of view as described previously), the additional spatial location data can be used to distinguish between similar spatial patterns occurring in different locations of the volume. This further assists in reducing false positives by differentiating coronary from non-coronary calcifications. For example, calcifications of the left anterior descending artery only occur near the anterior interventricular groove and the CNN leverages the information embedded in the spatial channels to incorporate this into the analysis. An ADAMAX optimiser was used along with a cross-entropy loss function against the categorical vessel classes.

### Validation and testing

Non-contrast prospective electrocardiogram-gated CT scan data from individuals aged ≥ 18 years who underwent assessment of CAC for investigation of CAD, where an Agatston CAC score was reported by level 3 expert readers, was included. Manual CAC scores were considered as ground truth. Scans were assessed for exclusion criteria a priori by the study investigators, and excluded scans were not included in the analyses. Exclusion criteria included high levels of noise, presence of multiple non-contrast series, missing data, and scans in individuals with coronary artery stents, coronary artery bypass grafts, prosthetic heart valves, permanent cardiac pacemakers, or other metal artefacts. In the dataset, multiple non-contrast CT series in an individual may have been present in cases where there was noise or acquisition issues. As such, these scans were excluded to reduce the potential for bias. The level of noise was determined by the signal-to-noise ratio in the descending aorta and the number of lesions (> 130 HU). The cut-off for excluding a scan due to noise was defined a priori through internal analyses as a signal-to-noise ratio of 1.7 with a maximum lesion number of 9000, such that no more than 5% of scans would be excluded.

For training and validation of the automated model, consecutive non-contrast-enhanced cardiac CT scans that were performed routinely for clinical purposes were retrospectively obtained from two separate institutions. The 3D CNN was trained on (1) 2251 Siemens Healthcare SOMATOM Force scans acquired from Envision Medical Imaging (Perth, Australia), with a validation set consisting of 735 scans that were randomly selected; and (2) 143 Philips Healthcare Brilliance 64 and 45 Toshiba Medical Systems Aquilion ONE scans acquired from University of Edinburgh (Edinburgh, Scotland), with a validation set consisting of 20 Philips Healthcare and 16 Toshiba Medical Systems scans.

The aorta model was trained using data from three separate CT scanners: Siemens Healthcare SOMATOM Force, Toshiba Medical Systems Aquilion ONE, and Philips Healthcare Brilliance 64. In total, 54 studies were used in the training set with seven studies in the validation set and 12 studies in the test set, all of which were randomly selected. A Dice score of 0.94 was achieved on the test set. A larger training set was used to develop the heart model with 94 studies in the training set across the three scanners, seven studies in the validation set, and 10 studies in the test set. The Dice score for the heart model was 0.86.

Following this, 2000 cardiac CT scans performed for clinical indications between December 2014 and May 2021 at the University of Ottawa Heart Institute (Ottawa, Canada) were retrospectively obtained. These CT images were acquired with standardised vendor-specific sequential scanning protocols, a tube voltage of 120 kVp and slice thickness of 2.5 mm (GE Healthcare) or 3 mm (Siemens Healthcare). After applying exclusion criteria, the automated CAC scoring model was tested on this independent dataset using images from the non-contrast series and CAC scores were computed for each scan. The graphic processing unit used was EVGA GeForce RTX3090 FTW3 ULTRA 24G (NVIDIA).

### Statistical analysis

Statistical analyses were performed using Python (Python Software Foundation). Data are presented as mean and standard deviation or count and percent. The total CAC score per individual was computed using the Agatston method and was further categorised according to the following five cardiovascular risk categories based on the score: 0, 1–10, 11–100, 101–400, and > 400.[3, 9] Automated CAC scores were compared to the manual and prospectively derived Agatston CAC scores as per the scan report (manual reference standard). Differences between proportions were compared using Pearson’s chi-square test. Correlation and agreement between methods were assessed using Spearman’s rank correlation coefficient (⍴), two-way intraclass correlation coefficient (ICC), Bland-Altman plots with mean difference and 95% limits of agreement, and Cohen’s linearly weighted kappa coefficient (*κ*). A two-tailed *p* value of < 0.05 was used to define statistical significance.

## Results

**Fig. 2:**
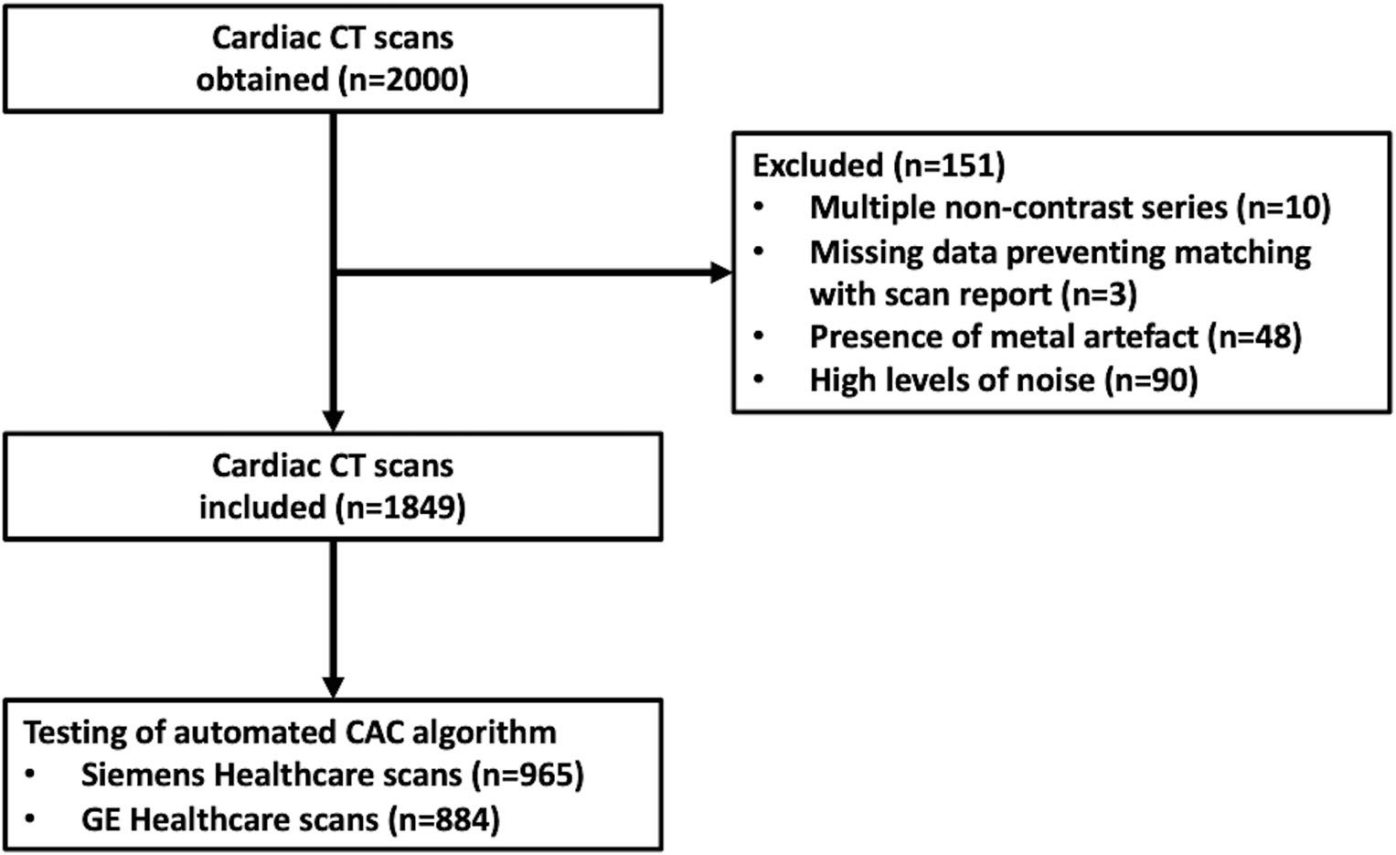
Study cohort of patients included for testing of automated CAC scoring algorithm**.** Abbreviation: CAC coronary artery calcium, CT computed tomography

Of the 2000 independent cardiac CT scans obtained, 151 were excluded and the remaining 1849 (92.5%) were used for testing the model as shown in Fig. 2. Of these 1849 scans, 965 (52.2%) were Siemens Healthcare Definition Flash and 884 (47.8%) were GE Healthcare Lightspeed VCT 64 slice scans. The mean age was 55.7 ± 10.5 years and 915 (49.5%) were males. The mean total analysis time per scan was 13.1 ± 3.2 s for the model, with variations in time due to the number of slices per CT scan required to analyse.

**Fig. 3:**
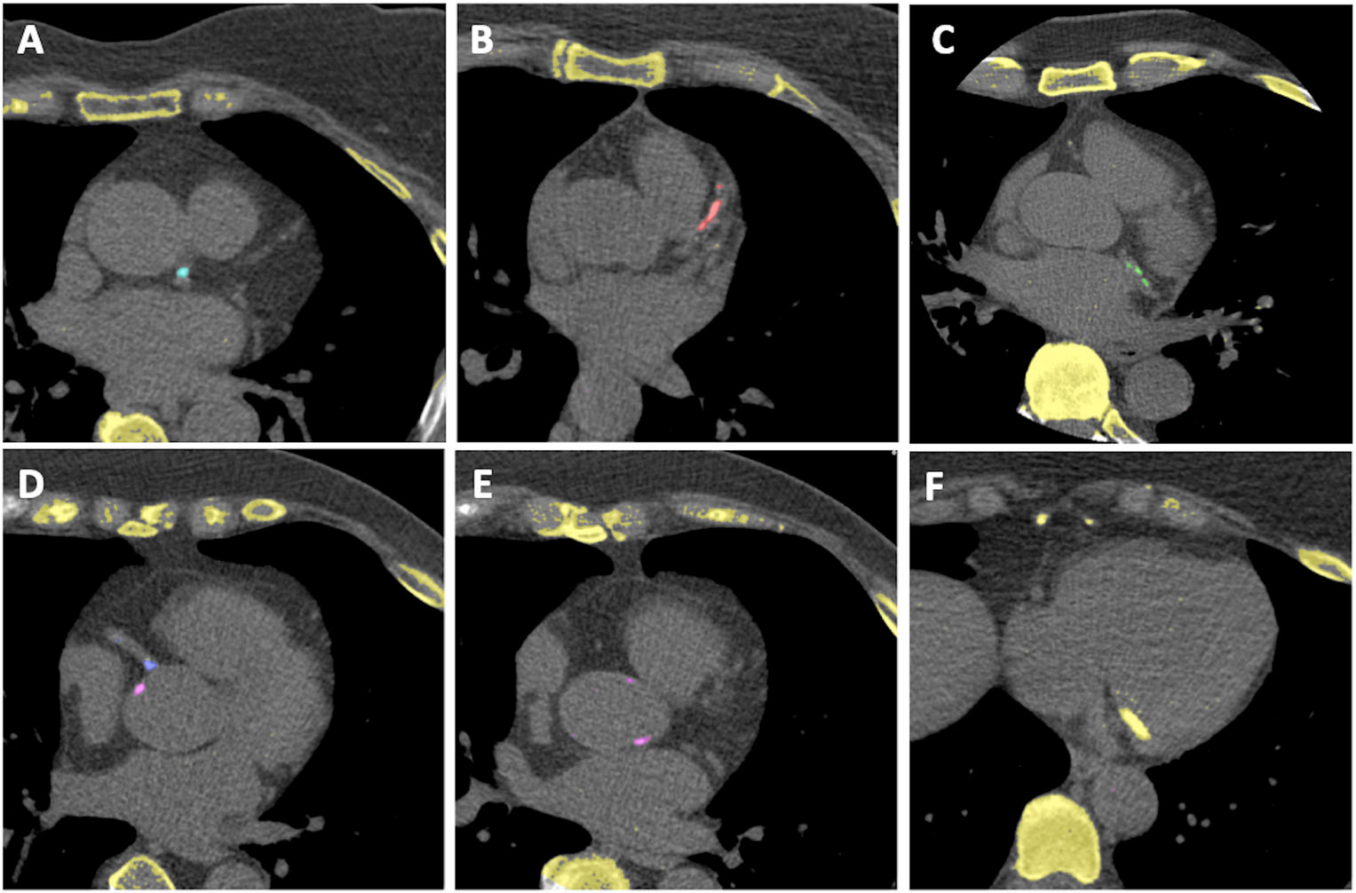
Example cases of calcifications detected. Figure legend: Successful detection of coronary artery calcification is seen in the (**A**) left main coronary artery, (**B**) left anterior descending coronary artery, (**C**) left circumflex coronary artery, and (**D**) right coronary artery. Successful detection of calcification that would not contribute to the calcium score is seen in the (**E**) aortic root and (**F**) mitral annulus.

Coronary artery calcifications were reported in 815 (44.1%) individuals with the reference standard and in 867 (46.9%, *p* = 0.09) individuals using the automated model. Examples of calcifications detected are shown in Fig. 3. Of the 1034 (55.9%) individuals with a zero CAC score with the reference standard, 92 (8.9%) had a positive score using the automated model (Table 1) and the reasons for this are detailed in Supplemental Table 1. Of the 815 (44.1%) individuals with a positive CAC score with the reference standard, 40 (4.9%) had a score of zero using the automated model (reasons detailed in Supplemental Table 1). A CAC score of > 100 Agatston units was reported in 274 (14.8%) individuals with the reference standard, of which 256 (93.4%) had a score of > 100 using the automated model. For CAC score > 100, the positive predictive value was 95.2% and negative predictive value was 98.9%. Of the 1575 (85.2%) individuals with a CAC score ≤ 100 Agatston units, 13 (0.8%) had a score > 100 using the automated model.

**Table 1:** Confusion matrix showing agreement between CAC scores derived by the manual reference standard and the automated model, based on Agatston score risk categories

Coronary artery calcium score		Predicted score using automated model					Total
		0	1–10	11–100	101–400	> 400	
Reference standard	0	**942**	71	18	3	0	1034
	1–10	30	**114**	7	0	0	151
	11–100	10	34	**336**	9	1	390
	101–400	0	0	18	**217**	0	235
	> 400	0	0	0	2	**37**	39
Total		982	219	379	231	38	1849

Bolded entries represent agreement in classification

Cohen’s kappa coefficient (*κ*) = 0.90, 95% CI 0.88–0.91, *p* < 0.001

Abbreviation: *CAC* coronary artery calcium, *CI* confidence interval

**Fig. 4:**
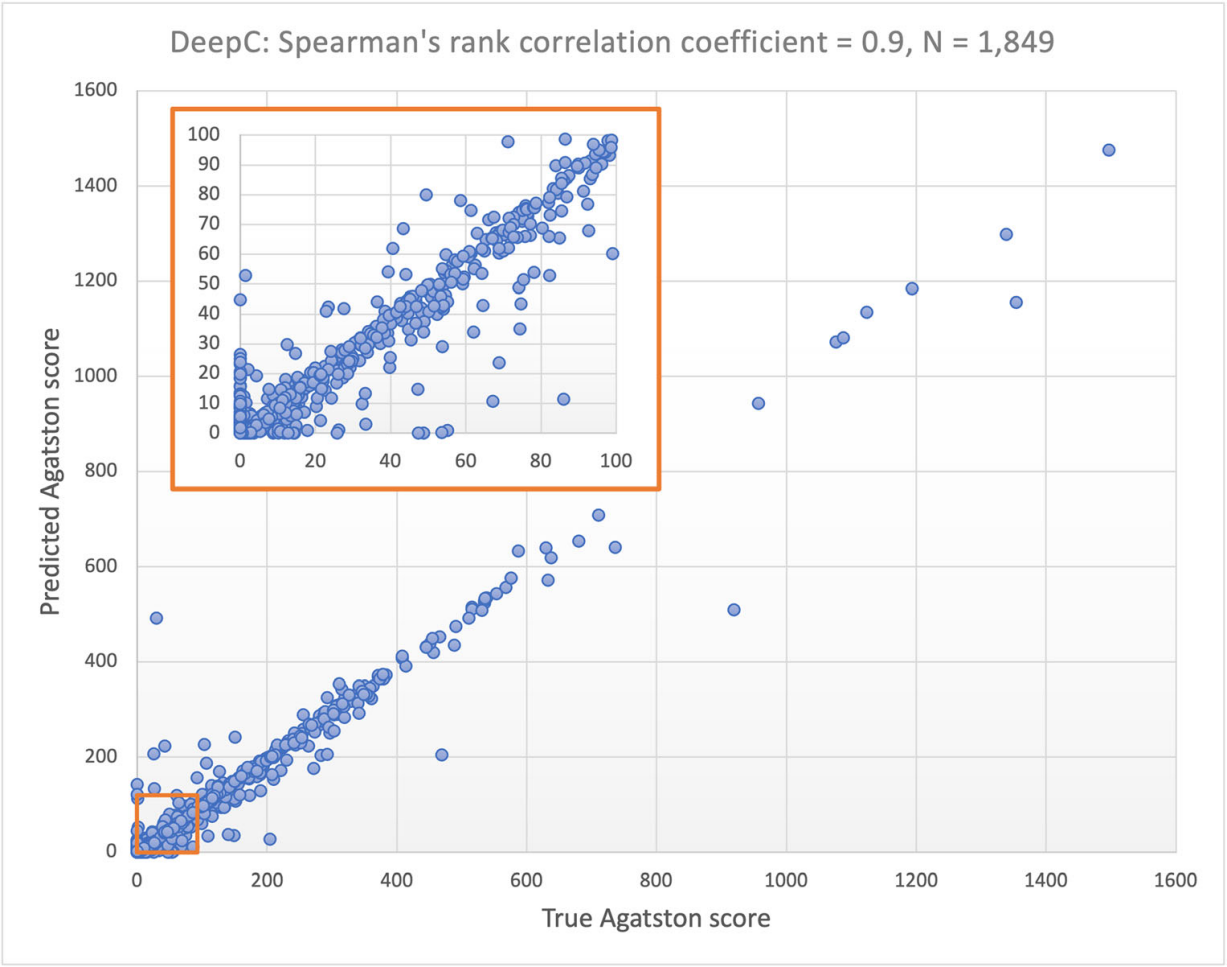
Scatter plot showing the correlation between CAC scores derived from the manual reference standard and the automated model. Spearman’s rank correlation coefficient (*r*) = 0.90, 95% CI 0.89–0.91, *p* < 0.001. Abbreviation: CAC coronary artery calcium, CI confidence interval

**Fig. 5:**
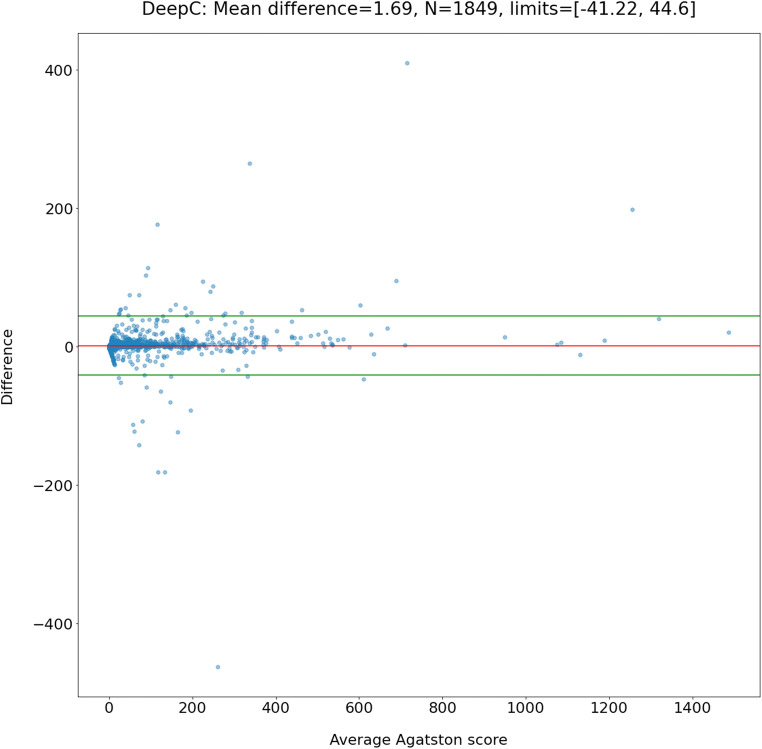
Bland-Altman plot showing agreement between the manual reference standard and automated model for CAC scoring. Mean difference = 1.69, 95% limits of agreement: −41.22 and 44.60. Abbreviation: CAC coronary artery calcium

The CAC score results from the automated model correlated very strongly (Spearman’s *r* = 0.90, 95% confidence interval [CI] 0.89–0.91, *p* < 0.001; and ICC = 0.98, 95% CI 0.98–0.99, *p* < 0.001) with the reference standard (Fig. 4). Bland–Altman analysis showed little difference in CAC scores between the reference standard and those predicted by the automated model, with a mean difference of 1.69 (95% limits of agreement: −41.22, 44.60) (Fig. 5). Overall, the fully automated model classified 1646 individuals (89.0%) into the same CAC score risk category as a reference standard. Of the 203 individuals that were reclassified, 171 (84.2%) were reclassified to the next risk category. When CAC score risk categories between the two methods were compared using Cohen’s kappa statistic (Table 1), there was almost excellent agreement (*κ* = 0.90, 95% CI 0.88–0.91, *p* < 0.001).

## Discussion

This paper presents a novel AI-based fully automated model that was developed to identify and quantify total per-patient CAC using non-contrast electrocardiogram-gated cardiac CT. The model demonstrated very high levels of correlation and agreement when compared with CAC scores obtained with the manual reference standard. Notably, the high accuracy of the model was maintained across the spectrum of cardiovascular risk categorisation and across different scanners. In addition, the model was able to compute the CAC score quickly, with a low analysis time per CT scan. These results lay the foundation for the translation of AI-based CAC scoring techniques in clinical practice.

The number of studies evaluating automated CAC scoring models is limited and comparison of studies is difficult due to differences in study design, methods, and CT datasets used. The present study is an extension of other similar studies with results that are in line with that of other cardiac CT studies evaluating automated methods, with high correlation and kappa coefficients of approximately 0.90 when compared to a human reference standard [12, 15, 16, 18–20]. Automated CAC scoring has also been the subject of notable publications over the past two years, including another multi-centre and multi-vendor study by Eng et al which included non-electrocardiogram gated CT scans [21, 23, 24]. AI models can also have a role in identifying and quantifying CAC in low-dose non-electrocardiogram-gated CT scans that include the heart, but which are performed for non-cardiac reasons such as lung cancer screening, thereby allowing opportunistic cardiovascular risk assessment [15, 16, 23–25]. However, given the reduced fidelity of non-gated chest CT, automated CAC score accuracies remain low (kappa coefficient in the range of 0.6 to 0.7) [20, 25].

A major challenge for automated CAC scoring methods is the ability to discriminate between true CAC and non-coronary calcification or noise without the need for human input. Some automated algorithms reported in the literature have relied on information from coronary computed tomography angiography (CCTA) slices to better define coronary artery anatomy, and therefore improve this discrimination [13, 26, 27]. However, CT images for quantifying CAC are not always acquired in conjunction with CCTA, which requires intravenous contrast. The focus of the present study is to achieve fast and accurate results exclusively on non-contrast electrocardiogram-gated cardiac CT scans. With this in mind, the present study developed an AI-based model using multiple CNNs designed to integrate 3D spatial location information, such that the position of the coronary arteries and aorta can be estimated, and lesions can be segmented without the need for contrast. The incorporation of a separate aortic segmentation model and a cardiac segmentation model may also help to reduce false positives by differentiating coronary from non-coronary calcifications. The model was also able to compute a CAC score in the order of seconds, which is substantially faster than manual methods and is an important prerequisite for implementing AI software [15, 17, 18, 20].

Another difference in the present study design is the use of five categories for the CAC score [3, 9]. Some studies using cardiac CT have assessed the ability of automated models to differentiate individuals into three or four CAC score risk categories [10, 19, 21, 26]. The inclusion of categories for CAC scores of 1–10 and 11–100 rather than one category for 1–100 assesses the added ability to identify individuals with minimal coronary artery plaque. Furthermore, individuals with a CAC score of zero have a very low 10-year cardiovascular risk and identifying these individuals can reduce the use of unnecessary preventative pharmacotherapies. [9] The automated model classified 9% of individuals with a reported CAC score of zero as having a positive score, and the reasons for this included detection of non-coronary calcification, noise, ground truth in question, and artefacts. On the other hand, only 5% of individuals with a reported positive CAC score were classified as having a score of zero using the automated model for similar reasons as above. Similar reasons have also been noted in previous AI studies in CAC scoring, thus highlighting an ongoing challenge [12, 28]. The majority of miscategorisation was between the 0 and 1–10 CAC score categories. From a clinical perspective, there are currently no recommendations for the use of a CAC score between 1 and 10 Agatston units to guide decision-making around preventive pharmacotherapies and thus the clinical impact of miscategorisation at this very low range of CAC score is reduced.

Although the CAC score is a continuous variable, guidelines recommend a CAC score of ≥ 100 Agatston units as a threshold for initiating statins [4–6]. The automated model accurately identified a CAC score >100 Agatston units in 95% of individuals compared with manual human assessment. On the other hand, < 1% of individuals with a CAC score ≤ 100 Agatston units had a score > 100 using the automated model. Although the model achieved respectable diagnostic performance, visual feedback can be provided to display regions contributing to the score and allow human operators to make modifications if required. Although not utilised in the present study, ambiguity detectors can be embedded to prompt feedback by human operators in cases of lesion uncertainty, which can resolve false positive or negative results and thus minimise the misclassification of CAC score categories [10, 12].

The use of AI in cardiovascular imaging has developed rapidly over the past decade [22, 23]. Fully automated models can minimise the physical and repetitive task of performing CAC identification, quantitation, and segmentation by reducing the need for human analysis. Re-direction of the human operator’s role to other tasks instead can further reduce costs associated with CAC scoring, thus making it more widely available. This is important as demand for CAC scoring is likely to increase substantially in the future due to guidelines now recommending its use as an additional non-invasive tool for cardiovascular risk-stratification [4–6]. Furthermore, automated models can be applied to large populations for screening or research purposes due to their ability to quantify CAC in seconds and because AI can potentially overcome measurement errors by human observers due to its high repeatability [21, 23]. Once fully validated, AI models for CAC scoring could also be used to train physicians in interpreting and reporting CAC scores.

The large number of CT scans used for training, validation, and testing of the automated CAC scoring model is a key strength of the present study. In addition, CT images in the dataset were acquired from several different scanners, reflecting the variability of real-world practice. Although CT scanners made by different vendors might produce different CAC scores, testing the automated model on a variety of CT images from different vendors or scanner types improves its applicability and generalisability to different practices [29]. A limitation of this study is that over half of the scans in the testing dataset had a zero CAC score. Although this is consistent with other studies and reflects the clinical practice and real-world populations, such a dataset would result in some cases where the model incorrectly reports a positive CAC score (particularly in the 1–10 CAC score category) [17]. Thus, the reported results could have been even better if not for this limitation. The accuracy of the model may therefore be reduced at very low levels of CAC and future iterations of the model will seek to refine this aspect. Furthermore, the model was not developed to quantify CAC per individual coronary artery, which may improve cardiovascular risk-stratification, but is not yet routinely performed for this purpose [8]. Scans with metal artefacts and high levels of noise were excluded as this can influence the CAC score, thus limiting the applicability of the automated model to such cases.

In conclusion, the presented fully automated AI-based CAC scoring model for cardiac CT is novel and rapid and shows high accuracy when compared to current manual CAC scoring methods. Future studies should evaluate its potential impact on clinical practice and workflow for cardiac CT reporting clinicians, as well as evaluate the association between automated CAC scores and patient outcomes.

## Supplementary information

ESM 1 (DOCX 21 kb)

## Abbreviations

AI: Artificial intelligence
CAC: Coronary artery calcium
CAD: Coronary artery disease
CCTA: Coronary computed tomography angiography
CNN: Convolutional neural network
CT: Computed tomography
HU: Hounsfield units
ICC: Intraclass correlation coefficient

## Declarations

### Guarantor

The scientific guarantor of this publication is Prof Girish Dwivedi.

### Statistics and biometry

One of the authors has significant statistical expertise.

No complex statistical methods were necessary for this paper.

### Methodology

• Retrospective

• Observational

• Multicentre
