# Supplementary material for: Evaluation of an artificial intelligence coronary artery calcium scoring model from computed tomography
Source: Eur Radiol. 2022 Aug 20;33(1):321–9. doi: 10.1007/s00330-022-09028-3 (PMC9755106; doi:10.1007/s00330-022-09028-3)
Supplement: Supplementary file 1 — (DOCX 21 kb) [file 330_2022_9028_MOESM1_ESM.docx]

**Supplemental Table 1 – False positive and negative results for zero calcium score**

| **Category** | **Reason** | **Percent** |
| --- | --- | --- |
| **False Positive**  AI *detected* calcium, but CACS zero with ground truth (n=92, 8.9%) | Non-coronary calcium (bone, aortic, mitral annulus) | 45% |
|  | Noise | 39% |
|  | Ground truth in question | 11% |
|  | Artefacts | 5% |
| **False Negative**  AI *did not detect* calcium, but CACS >0 with ground truth (n=40, 4.9%) | Misclassification* of coronary calcium | 45% |
|  | Partial misclassification* of a lesion | 33% |
|  | Noise (mainly affecting distal RCA) | 20% |
|  | Ground truth in question | 3% |

Abbreviations: AI = artificial intelligence, CACS = coronary artery calcium score, RCA = right coronary artery

* As the algorithm classifies individual voxels, it is possible for 2 connected voxels to have different classifications. This is by design so that lesions that span the aorta/left main coronary artery and left anterior descending artery/circumflex artery boundaries can be classified.
